# Psychometric Properties of the Video Game Experiences Questionnaire (CERV), Problematic Use of Video Games and the Link with the Use of Mobile Devices in Mexican Children

**DOI:** 10.3390/ijerph22040476

**Published:** 2025-03-23

**Authors:** Rocío Martínez-Hernández, Jorge Zamarripa, Georgina Mayela Núñez Rocha

**Affiliations:** 1 Facultad de Organización Deportiva, Universidad Autónoma de Nuevo León, San Nicolás de los Garza 66455, Mexico; rocio.martinezhe@uanl.edu.mx; 2 Facultad de Salud Pública y Nutrición, Universidad Autónoma de Nuevo León, Monterrey 66455, Mexico; georgina.nunezrc@uanl.edu.mx

**Keywords:** video games, addiction, problematic use, questionnaires, validation, psychometrics, self-report

## Abstract

When the use of video games is inappropriate in terms of time and content, it becomes a health risk. The objective of the present study was to analyze the psychometric properties of the Video-Game-Related Experience Questionnaire (CERV), determine its problematic use and know the link between the use of mobile devices (MD) and Mexican children. Methods. The study followed an instrumental and comparative design, with *n* = 519 children. Of these, 61.5% were from Jalisco, and 38.5% were from Nuevo Leon. The sample consisted of 50.1% girls, with 39.7% and 33.7 were in sixth and fifth grade of primary school, respectively. The mean age of participants was 10.50 ± 0.94 years, with ages ranging from 9 to 13 years. In addition, 86.7% of children had access to a DM, 45.3% of children who had a DM used it to play, and 59.0% exceeded the recommended usage time of more than two hours. The Video-Game-Related Experience Questionnaire was used. For the factorial structure, a confirmatory factor analysis was conducted using the Diagonal Weighted Least Squares (DWLS) estimation method. The goodness-of-fit indices were as follows: chi-square value over degrees of freedom (*X*^2^/*gl*), CFI, NNFI, and RMSEA. Results. The goodness-of-fit indices were shown as follows: *X*^2^/*gl* = 1.16; RMSEA = 0.018; SRMR = 0.048; CFI = 0.99; TLI = 0.99; NNFI = 0.99. Acceptable reliability was found with both Cronbach’s alpha and McDonald’s omega greater than 0.80. Furthermore, 41.6% of participants had potential or severe problems with video game use, and the use of mobile devices (DMs) was significantly associated (*p* < 0.001) with potential or severe problems. Conclusion. It is suggested that educational programs be implemented regarding the consequences of excessive video game use, the usage of DMs, and the importance of restrictive use per se for parents.

## 1. Introduction

The development and expansion of Information and Communication Technologies (ICT) in modern society has opened a huge panorama of possibilities for the use of video games and mobile devices (MDs), producing a change in lifestyle, especially in children and adolescents [1,2,3,4,5,6,7]. 

In Mexico, the National Survey on Availability and Use of Information Technologies in Homes (ENDUTIH) indicated that 75.6% of the population aged six years or older used the internet, which revealed an increase of 4.1 percentage points compared to the 2020 survey (71.5%) [8]. The report added that the use of hours on the internet and access to mobile devices in this population (children six years or older) tends to increase compared to that in previous years and added that 77.3% of Mexican children exceeded the time limit they should spend in front of a screen or mobile device (more than two hours) [2,8,9].

On the other hand, Statista’s Digital Market Outlook reported that Mexico is one of the five countries with the most gamers worldwide, ranking fifth, which means that more than 50% of the Mexican population uses video games [10]. Various studies reported [11,12,13,14] report that the use of video games could have increased since the confinement imposed by the World Health Organization (WHO) due to the SARS-CoV-2 pandemic derived from the need to create social ties that humans have. 

The above statistics generate a certain degree of concern within the scientific community, mainly in the health area, since the use of video games and mobile devices (MDs) often have an addictive capacity; prior to addiction, it is important to mention that problematic use per se occurs; we speak of problematic use of video games or mobile devices when their use is not appropriate in terms of time and content [1,12,14,15,16,17,18]; problematic use of video games is considered as a persistent need to play video games for prolonged periods (periods of more than 2 h), causing a risk for the development of mental disorders or negative effects on psychological, social, and physical health, affecting certain areas of the lives of children and adolescents [2,12,16,17,19].

When problematic use of video games or mobile devices is not controlled or attended to appropriately and early, it could become a new form of addiction, which is why it is currently considered one of the new addictions that is not related to the use of harmful substances such as alcohol and drugs [16,18]. Addiction is defined as excessive and uncontrollable behavior, which physically and psychologically destroys the individual. It leads to the displacement of important actions (family, responsibilities, friends, among others) despite the negative consequences for both the person and those around him. The main symptoms of addiction are mood disorders, withdrawal syndrome, conflict, and relapse [4,20,21]. For the above reasons, some years ago, internet gaming disorder was incorporated into Section III of the DSM-5. Although it suggests that more scientific evidence is needed to consider it as an addiction and include the use of video games as an addiction, within the scientific and public health community it is known that its prevalence and use of these continues to increase and will not stop [20,21].

Video games are games played through electronic devices (console, computer, or mobile devices). They are considered a new form of leisure, recreation, fun, and entertainment, widely known by children, adolescents, and even adults [6,11,15,16]. In the peculiar condition of addiction and attraction that video games have, several factors intervene, such as the incitement to curiosity, long-term concentration on the narrative, rewards or punishments when playing or using the game, control over the virtual world which could not be accessed in the real world, empathy with the characters, and live interaction and competition with other players in the case of online games [12,16,18,19,22].

In addition to the above characteristics, the “uses and gratifications” approach explains how video games promote the satisfaction of some social and psychological needs in users, that is, video games have the unique ability of modulating the mood through pleasurable sensations (dopamine secretion). As a result, when playing, they are released or temporarily forget unpleasant situations, which they cannot control in the real world. However, it is important to mention that the individual psychological and social state can be decisive in determining whether video games become problematic leisure activities. This holds true regardless of age or gender, since so far there has been no conclusive scientific evidence to suggest that these factors influence the problematic use of video games [12,18].

Other conditions that cause or encourage the use of video games to become an addiction are the high availability of electronic devices or mobile devices without control or supervision by an adult [16,23], limited or no opportunities to do recreational or leisure activities such as living in very small houses, reliance on social assistance, and lack of safe parks, need to interact with peers who have interests in video games, having learning problems, unpleasant moments, poor coping activities at school, and lack of knowledge about other leisure activities [16].

Regardless of the conditions that encourage the use of video games, their existence and the upward trend of their use are tangible facts [2,7,8,11], as well as the addictive potential they have [15,16] and consequently the harmful effects on children’s health. These effects include decreased intra-family communication, increased symptoms of depression, increased desire to be alone, decreased relationships with peers, false perspectives of the future, a bad attitude, social withdrawal, and introversion [7,12,16].

Negative effects on psychomotor development and decreased academic performance are also mentioned [14,15], as well as problems of overweight and obesity and their subsequent consequences such as hypertension and insulin resistance [7,9,24]. Another study reports positive and significant correlations between the frequency of use of video games (Fortnite, Free Fire, and Call of Duty) and aggressive behaviors, as well as between the time spent on video games and proactive aggression [25]. Other researchers point out that problems related to excessive use of phones or mobile devices are similar to problematic use of video games [5,26,27].

In parallel, some other studies indicate that the use of video games can help reduce sedentary lifestyle levels [28,29], promote more playful learning on different health topics, obtain motivational and emotional benefits and improve creativity [17,30]. Benefits have been observed in physical activity and executive function in children with attention deficit hyperactivity disorder (ADHD). However, it is important to note that the average time spent on video games was 30–60 min, 3 to 4 times a week [31,32,33]. Although video games are not harmful when the time spent using them is less than two hours a day, excess use can lead to negative effects [2,8,9].

In Mexico, there are several studies that have evaluated the use of screens (television, video games, and telephones), which have focused on knowing the time of use [3,9,13,24], addiction to social networks [34], and the problematic use of video games in high school students, adolescents, and adults [11,12,18,25,35]. Few studies have focused on school-age children, such as the one carried out by Arellanez et al. [22] and Dorantes-Argandar & Molina-Vega [35]. The latter author did not report the validation of the instrument used, only the reliability through Cronbach’s alpha. It highlights that this population group has problems with the use of video games and DMs, which leads to the need to do this type of studies, in order to have reliable tools that help evaluate this type of problems and to be able to intervene in health matters before they become an addiction problem. It is also important to note that the latent consumers of video games are children and adolescents and that the DSM-5 criteria to identify their problematic use of online games are focused on the adult population and not focused on children or adolescents, these being the ones who present the greatest risks [4,21].

The purposes of the present study are as follows: (1) to analyze the psychometric properties of the Video-Game-Related Experience Questionnaire (CERV) adapted to the context and in Mexican children; (2) to determine the problematic use of video games; and (3) to know the link between the use of mobile devices and Mexican school-aged children.

## 2. Material and Methods

### 2.1. Participants

The study with an instrumental and comparative design [36] included 541 children from seven primary schools participated in two different states of the Mexican Republic: five from the West (the State of Jalisco) and two from the Northeast (the State of Nuevo Leon). Twenty-two surveys were excluded due to incomplete information, leaving a final sample of children (*n* = 519). Of the total sample, 61.5% of the children were from Jalisco, 38.5% were from Nuevo Leon, 50.1% were girls, 39.7% and 33.7% were in sixth and fifth grade of primary school, respectively, and 37.8% of the children were 11 years old, with a mean age of 10.50 ± 0.94 years ranging from 9 to 13 years; 86.7% of children had access to a mobile device, 45.3% of children who had a mobile device used it to play, and 59.0% of children used the mobile device for more than two hours.

The inclusion criteria for the sample required children to obtain the informed consent from their legal guardians and to agree to give their assent. Children who had any disease (asthma, diabetes, bronchitis, among others) were excluded since it could limit the realization of some outdoor activities, and those children who had a learning delay were also excluded since these children might not have a full understanding of the questions. This was evaluated by directly asking the teacher in charge of the group and the parents or legal guardians.

For the sociodemographic collection of the data, an ad hoc instrument was designed that included questions about the school grade (fourth, fifth, or sixth grade of primary school), gender (boys or girls), age in completed years, access to mobile devices (personal or those of a guardian), reason for using mobile devices (playing, studying, talking to friends or family, and other reasons such as making videos, using tik tok, and engaging with social networks), and approximate hours of use of mobile devices (less than 1 h, 2 h, 3 h, 4 h, or 5 h or more). The sample selection was non-probabilistic for convenience since children were selected according to accessibility [37]. 

### 2.2. Instrument

The Spanish version of the Video-Game-Related Experience Questionnaire (CERV, Table S1) was used in children from seven primary schools in two different states of the Mexican Republic, aged between 9 and 13 years. The CERV is an instrument used to identify problematic use of video games in Spanish adolescents. It was created by Chamarro et al. [15] and designed using two questionnaires: the Internet-Related Experiences Questionnaire known as CERI and the Mobile-Related Experiences Questionnaire, abbreviated as CERM [38]. The CERV consists of 17 items and is composed of two factors: the first is psychological dependence (such as questions about problem avoidance, loss of control, mood swings, and focus); the second factor is called negative consequences of video game use (such as decreased academic performance, difficulty having social relationships, conflicts with parents or adults, and agitation). The questions have a four-point Likert-type response option (never, sometimes, quite a few times, and always). The higher the score, the greater the problem with video game use. The maximum score is 68 points, and the minimum is 17 points [15].

### 2.3. Procedure

Permission was requested from the zone supervisor of the educational institutions to present the project to the directors of five schools in the southern coastal region of the State of Jalisco. In the educational institutions where the directors agreed to participate in the project, the informed consents were delivered and signed by the parents or legal guardians.

Once the signatures of the children’s guardians on the informed consent were obtained, the principal investigator and two graduate students appeared before the group to inform the children about the objective of the study, voluntary participation, the emphasis on the fact that it was not an exam—there were no right or wrong answers. They highlighted that it would not affect their academic performance if they did not wish to participate and could stop answering the questionnaire at any time and that only the researchers would know the results of their answers. Finally, the children who agreed to participate and obtained the consent from their parents or guardians were asked to give their consent to complete the questionnaire.

The children answered the questionnaire in the classroom, always accompanied by the principal investigator who resolved any questions that might arise at the time of application. The response time ranged between 20 and 35 min. This same procedure was carried out with the children from the State of Nuevo León.

This research project complied with the provisions of the General Health Law on Health Research, published by the Official Journal of the Federation in 2014 [39] and the Declaration of Helsinki of the World Medical Association (WMA) [40]. Therefore, this research project was submitted to the Research Ethics Committee of the Faculty of Sports Organization (CEIFOD) with registration CONBIOETICA-19-CEI-002-20220418, which granted its approval with registration code CEIFOD 0124 015.

### 2.4. Data Analysis

To check the factorial structure of the questionnaire, a confirmatory factor analysis (CFA) was performed. Considering the ordinal nature and the sample size, the Diagonal Weighted Least Squares (DWLS) estimation method was used. The goodness-of-fit indices used to analyze the adequacy of the data were as follows: the chi-square value on degrees of freedom (*X*^2^/*df*), the CFI, the NNFI, and the RMSEA. Taking as a reference the guidelines of Batista-Foguet et al. [41], the goodness-of-fit index of the model was taken as an *X*^2^/*df* ratio of less than 4, CFI and NNFI values above 0.90 were considered to have an acceptable fit; in the case of the RMSEA, values equal to or less than 0.08 were considered satisfactory.

A descriptive analysis was performed for the sociodemographic characteristics, frequencies and percentages for categorical variables, and means and standard deviations for continuous variables. To determine a classification for problematic video game use, a non-hierarchical cluster analysis (K-means) was performed with three categories: low (12–22 points), medium (23–30 points), and high (31–54 points). For data analysis, the statistical package, IBM Statistical Package for the Social Sciences (SPSS version 21 for Windows), was used, and for the CFA, the software JAPS Version 19 was used.

## 3. Results

### 3.1. Confirmatory Factor Analysis (CFA)

The CFA was performed with the 17 items proposed by Chamarro et al. [15]. The goodness-of-fit indices of the two-factor structure (dependence and avoidance and negative consequences) were shown as follows: *X*^2/^*gl* = 1.16; RMSEA = 0.018; CFI = 0.99; NNFI = 0.99 [41], which indicated that the proposed model had an acceptable fit in the Mexican child population. The standardized factor loadings of all the items analyzed reached significant values (*p* < 0.001) (see Table 1).

The covariance between the factors showed an estimate of 0.99 (*p* < 0.001), so it was considered to continue with the names of the domains proposed by Chamarro et al. [15]: dependency and avoidance (D&E) and negative consequences (NCs), with 8 and 9 items. respectively.

### 3.2. Reliability Analysis

The internal reliability values were analyzed using Cronbach’s alpha and McDonald’s omega, and it was observed that both the dimensions and the global scale had acceptable reliability with Cronbach’s alpha and McDonald’s omega values higher than 0.80 [42] (see Table 2).

### 3.3. Cluster Analysis

To determine the problematic use of video games, a K-means cluster analysis was performed with three categories. Considering that the higher the score, the greater the problematic use, the analysis suggested the following classification: the first group (*n* = 303; 58.4%) with the lowest scores (between 12 and 22 points) was categorized as having no problems with the use of video games (SP), the second group (*n* = 161; 31%) with medium scores (between 23 and 30 points) was categorized as having potential problems with the use of video games (PP), and the third group (*n* = 55; 10.6%) with the highest scores (between 31 and 54 points) was categorized as having severe problems in the use of video games (PS).

When evaluating the problematic use of video games by gender, it was observed that boys had a higher prevalence of PP and PS, with 18.9% and 8.1%, respectively, compared to girls, with significant differences by gender (*p* < 0.001; see Table 3).

When the problematic use of video games was compared by state of the Mexican Republic, it was observed that there was a higher proportion of children from the State of Jalisco who presented PP with the use of video games compared to children from Nuevo León, with 20.6% and 10.4%, respectively. However, no significant differences were found by state of the republic (*p* > 0.05; see Table 3).

Finally, the problematic use of video games was compared by hours of use of mobile devices. The results showed that children who used mobile devices for more than two hours presented PP and PS of video game use, with 20.4% and 8.9%, respectively, compared to those who used mobile devices for less than two hours, with 10.6% and 1.7%, respectively, showing significant differences (*p* < 0.001; see Table 3). 

## 4. Discussion

The present study had three objectives. The objective was to analyze the psychometric properties of the Video-Game-Related Experience Questionnaire (CERV) adapted to the context and in Mexican children, previously reported by Chamarro et al. [15], the second objective was to determine the problematic use of video games, and the third objective was to know the link between the use of mobile devices and Mexican school children.

Regarding objective one, the results showed that the structure of the two-factor model of CERV proposed by Chamarro et al. [15] is adaptable to the Mexican school population with acceptable CFI, NNFI, and RMSEA values and significant values of the standardized factor loadings of all items. The questionnaire also presents an acceptable internal reliability both in the dimensions and in the global scale with Cronbach’s alpha and McDonald’s omega values higher than 0.80 [41]. It is important to mention that in the present study, the standardized factor loadings of all items had acceptable values but were slightly lower than those reported by Chamarro et al. [15], which could be explained by the smaller sample size compared to that used by Chamarro et al. [15]. Another reason could be explained by the parameter estimation model that was used. In the present study, the Diagonal Weighted Least Squares (DWLS) estimation method was used, and it is unknown which method was used by Chamarro et al. [15]. However, it is important to note that with the exception of item two, all factor loadings are higher than 0.50, which is considered strong [43]. These results show that further studies should be conducted to confirm the validation of the instrument in the Mexican school population. Although the first results show an adequate adaptation, more studies are needed to accurately identify children over 8 years old who have problems with video games.

Regarding the second objective, nearly half of the study population has PP or PS with the use of video games. These results are consistent with those reported by Arellanez et al. [22], where half of the population has PP or PS problems. In addition, it can be observed that although there is a high prevalence of these problems in the Mexican child population, this prevalence of PP or PS problems is lower than that reported by Chamarro et al. [15], where nearly 80% of adolescents have PP or PS with video games. These findings contrast with those reported by Portillo-Peñuelas et al. [18] and Rehbein et al. [44], where a low percentage of adolescents have problems with video games. The consistent results show that we are facing a latent problem with problematic use of video games in the population of children and adolescents. Although the proportion of Mexican children who have problems with video games is lower than that observed in the Spanish adolescent population, it is important to note that if the Mexican adolescent population were evaluated, we would encounter similar findings to those of Spanish adolescents. On the other hand, the discrepant results could be due to the instrument used. While in this study only the problematic use of video games was evaluated, Portillo-Peñuelas et al. [18] and Rehbein et al. [44] report that they evaluated video game addiction, not problematic use, which are different conditions.

Regarding the third objective, it was observed that almost all children have access to some DMs, either their own or that of a family member. Likewise, it was observed that almost half of the children use the mobile device to play, and more than half use the mobile device for more than four hours. These results agree with those reported in the National Survey on Availability and Use of Information Technologies in Homes [8], as reported by Elhai et al. [26], Jaimes et al. [2], and Shuai et al. [14] where it is mentioned that most people, including children, have access to DMs and exceed the time limit that they should spend in front of a screen. However, these findings differ from what was reported by Soltero et al. [9] and Lajous et al. [24], who indicated that children spend less than 2 hours a day playing and that only a small part of the population exceeds the time limits in front of the DMs. These discrepancies could be explained by the way of extracting the information, since the information reported by Soltero et al. [9] and Lajous et al. [24] was extracted by the parents, that is, it was the parents or guardians of the children who provided the information on the time that the children spent on the DMs. This could present a bias in the information since in the Mexican culture, both parents work, which limits constant supervision of the use of children’s video games and DMs, increasing the risk of overestimation or underestimation [14,21].

It is important to note that these findings should be a focus of attention for health personnel and parents since prolonged use of DM is associated with the use of video games, lack of concentration in school activities, and interference with the performance of recreational activities that could improve attention span. Recent research indicates that interactivity with DMs could contribute to the development of symptoms of attention deficit hyperactivity disorder (ADHD) [14,45]. In addition, it is important to keep in mind that it is not DMs that cause problematic use, but rather the applications within DM conthat make it a risk factor [21].

Regarding the prevalence of problems with the use of video games by sex, in the present study, it was found that boys have a higher prevalence of PP and PS with video games compared to girls. In contrast, a low percentage of girls had PP or PS, and nearly a third of girls do not have problems with video games. These results are consistent with those reported by Rehbein et al. [44], Desai et al. [46], Portillo-Peñuelas et al. [17], and Lloret Irles et al. [44], where boys present more problems with the use of video games. This could suggest that boys are more prone to having problems with video games than girls, which contrasts with what was reported by Arellanez et al. [22] and Chamarro et al. [15], where girls presented a higher prevalence of problems with video games. Similar results could indicate that when one goes from childhood to adolescence, there is an inclination for girls to develop problematic use of video games. However, it is important to continue investigating this relationship between video games and sex since the age groups in the studies compared are heterogeneous, and there is no homogeneity in the measurement scales, which could be biasing the results reported and contrasted.

Continuing with the third objective, the results of the present study showed that the hours spent by children on DMs are significantly associated with video game problems. These results are similar to those reported by Muñoz-Miralles et al. [5], where people who spend more hours playing DMs could present PP and PS with the use of video games. However, these results contrast with what was reported by André et al. [20] and Carbonell [21], who report that the time spent playing is irrelevant; what matters are only the negative consequences that can be derived from it and the ability to control oneself. It is important to point out that the time that the child spends playing video games or DMs prevents him from carrying out other activities, such as peer interaction and sports activities which promote optimal physical, social and psychological development [21]. In parallel, regarding the place of origin, in the present study, no significant differences were found with the problematic use of video games, which is in agreement with that reported by Portillo-Peñuelas et al. [18], where no differences were found with the problems of addiction to video games by geographical area (urban and suburban). This supports that access to both mobile devices and video games is bridging the geographical gaps and is expanding rapidly.

One of the strengths of this study is the inclusion of children from different geographic areas of the Mexican Republic. Another additional strength would be the type of instrument that is being validated. In this case, self-reporting is a way in which children relate situations that have occurred due to the use of video games, rather than relying on reports given by parents. This is important to consider, since in the current context, most parents work, so they are not at home all the time and cannot supervise the activities that the child performs with DMs or electronic devices. It should be added that most of the evaluations that have been carried out in Mexico are about screen time and overlook to the problematic use of video games. Therefore, the findings of the study provide an overview of the context of video game use among Mexican children from 9 to 13 years old, to continue this line of research.

It is important to mention that, although the present study meets the sample size suggested to perform a CFA, it has several limitations. One of them is that the sample size required to perform a factorial invariance analysis was not met. Therefore, it would be important for future research to overcome the limitations of the present study. It is advised that future studies increase the sample size to perform factorial invariance analysis, as well as convergent and discriminant validity, and use the test-retest method, in order to have greater precision of the questionnaire. Additionally, it is important to mention that this instrument only evaluates problematic use of video games and does not address the positive or negative experience with the use of video games or addiction per se. Furthermore, it should be kept in mind that the self-report instrument could only be used for children over 7 years of age, since reading comprehension could be limited in younger children. It would also be important to evaluate the agreement between the children’s self-report and the reports provided by parents or guardians. Finally, it is important to note that both problematic use and addictions to video games and DMs are practically new topics and represent a great challenge for clinicians, researchers, and public health personnel, because, in addition to identifying the symptoms and risk behaviors associated with this type of problems, there are different types of video games and their constant evolution, social pressures, and the need for adaptation.

## 5. Conclusions

Overall, the CFA with the 17 items presents an adequate fit in the Mexican child population with an acceptable internal reliability by dimension. However, it is recommended to continue with the validation of the instrument through a factorial invariance analysis, a convergent and discriminant validity to have a more solid validation of the instrument.

Based on the results found, almost all children have access to a DM. Half of the children use a phone to play, more than half of the population uses the DM for more than two hours, exceeding the allowed limit. Just over half of the children present PP or PS with the use of video games. Finally, the problematic use of video games was significantly associated with sex and the number of hours spent using a DM [26,27]. Hence, the importance of carrying out this type of studies lies in its ability to identify risk factors at an early age. Considering that the development and access to this type of technology is inevitable, it is therefore necessary to begin to understand the context of these public health problems.

In light of the above, it is suggested that educational programs be implemented on the consequences of excessive use of video games and DMs. Therapy and education programs should be introduced for parents or guardians, emphasizing the importance of restrictive use of DMs. While video games are often used for recreation or fun, the psychological problems and the negative consequences that arise due to excessive use of them significantly affect children, because they are at a pivotal stage in their development, where they begin to form social relationships with their peers and create healthy lifestyle habits.

The present research study only investigated problematic video game use and did not focus on what types of video games are most harmful to health or the types of experiences children have when using them. It is, therefore, suggested that further research investigate the types of video games and the experiences that encourage children to use video games in an uncontrolled manner.

## Figures and Tables

**Table 1:** Standardized factor loadings of the Video-Game-Related Experience Questionnaire (CERV).

Questionnaire Items	Factor Loadings	*M*	*SD*	Skewness	Kurtosis
Dependence and Evasion (D&A)					
1. To what extent do you feel restless about issues related to video games?	0.563	2.11	1	0.577	−0.821
2. When you are bored, do you use video games as a form of distraction?	0.456	2.66	1	−0.103	−1.302
3. How often do you stop what you are doing to spend more time playing video games?	0.674	1.91	1	0.846	−0.469
8. When you have problems, does using video games help you forget about them?	0.697	1.94	1	0.795	−0.677
10. Do you think that life without video games is boring, empty, and sad?	0.662	1.74	0.98	1.169	0.189
11. Do you get angry or irritated when someone bothers you while you are playing a video game?	0.687	1.94	1	0.825	−0.553
15. Do you downplay the amount of time you have been playing video games?	0.633	1.80	1	1.010	−0.217
16. Do you stop hanging out with your friends to spend more time playing video games?	0.581	1.54	0.94	1.603	1.262
**Consecuencias Negativas (CN)**					
4. Have your friends or family criticized you for spending too much time and money on video games or told you that you have a problem, even though you think it is not true?	0.596	1.81	1	1.0047	−0.186
5. Have you been at risk of losing your friends, a homework meeting, a school assignment, and academic opportunities because of video game use?	0.525	1.54	0.93	1.615	1.241
6. Do you think that you have stopped paying attention in class and have had lower grades because of video game use?	0.521	1.80	1	1.062	−0.139
7. Do you lie to your family or friends about the frequency and length of time you spend on video games?	0.588	1.63		1.434	0.868
9. How often do you ignore annoying thoughts about your life and replace them with pleasant thoughts about video games?	0.727	1.97	1	0.748	−0.795
12. Do you suffer from sleep disturbances (falling asleep late, losing sleep) due to aspects related to video games?	0.605	1.64	0.97	1.3335	0.505
13. When you are not playing video games, do you feel agitated, worried, or sad?	0.631	1.58	0.95	1.503	0.997
14. Do you feel the need to spend more and more time on video games to feel satisfied?	0.648	1.66	0.96	1.323	0.565
17. When you play video games, does time go by without you realizing it?	0.674	2.36	1	0.280	−1.392

Note: Own elaboration, extracted from a direct survey. *M*—average; *SD*—standard deviation.

**Table 2:** Descriptive statistics and reliability for the CERV * subscales.

Dimensions	*N*	*M*	*SD*	Min.	Max.	*A*	Ω
Negative consequences (CNs)	519	15.99	6.03	9	46	0.83	0.83
Dependency and evasion (D&E)	519	15.64	5.45	8	32	0.81	0.81
CERV global	519	31.64	10.9	17	68	0.90	0.90

Note: *N*—the number of the sample; *M*—mean; *SD*—standard deviation; *α*—Cronbach’s alpha; Ω—McDonald’s omega; * CERV—Video-Game-Related Experience Questionnaire.

**Table 3:** Categorization of problematic use of video games based on gender, state of the republic, and time of use of DMs *.

Variables		SP	PP	PS	*p*
		%	%	%	
Gender	Boys	22.9(119)	18.9(98)	8.1(42)	0.001
	Girls	35.5(184)	12.1(63)	2.5(13)	
State of the republic	Jalisco	34.5(179)	20.6(107)	6.4(33)	0.29
	Nuevo León	23.9(124)	10.4(54)	4.2(22)	
Time of DM use	Two hours or les	28.7(149)	10.6(55)	1.7(9)	0.001
	More than two hours	29.7(154)	20.4(106)	8.9(46)	

Note. Own elaboration; data extracted from direct survey. * DMs—mobile device; SP—no problems; PP—potential problems; PS—severe problems; *p*-values of < 0.05 were considered statistically significant.

## Data Availability

The data supporting the findings of this study are available from the corresponding author, J.Z., upon request and with reasonable justification.

## Supplementary Materials

The following supporting information can be downloaded at: https://www.mdpi.com/article/10.3390/ijerph22040476/s1, Table S1: Cuestionario de Experiencias Relacionadas con los Videojuegos (CERV) adaptado al contexto mexicano para niños de edad escolar.
