# Supplementary material for: Psychometric Properties of the Video Game Experiences Questionnaire (CERV), Problematic Use of Video Games and the Link with the Use of Mobile Devices in Mexican Children
Source: Int J Environ Res Public Health. 2025 Mar 23;22(4):476. doi: 10.3390/ijerph22040476 (PMC12026956; doi:10.3390/ijerph22040476)
Supplement: Supplementary file 1 [file ijerph-22-00476-s001.zip › ijerph-3467407-supplementary.pdf]

Tabla S1: Cuestionario de Experiencias Relacionadas con los Videojuegos (CERV) adaptado al contexto mexicano para niños de edad escolar

	Respuestas			
**Ítems del instrumento**	**Nunca**	**A veces**	**Bastantes veces**	**Siempre**
1. ¿Hasta qué punto te sientes inquieto por temas relacionados con los videojuegos?				
2. ¿Cuándo te aburres, usas los videojuegos como una forma de distracción?				
3. ¿Con que frecuencia abandonas lo que estás haciendo para estar más tiempo jugando a los videojuegos?				
4. ¿Te han criticado tus amigos o familiares por pasar mucho tiempo y gastar dinero en los videojuegos o te han dicho que tienes un problema, aunque creas que no es cierto?				
5. ¿Has tenido el riesgo de perder a tus amigos, una reunión de tarea, un trabajo de la escuela y oportunidades académicas por el uso de los videojuegos?				
6. ¿Piensas que has dejado de poner atención en clases y bajado de calificaciones por el uso de los videojuegos?				
7. ¿Mientes a tus familiares o amigos con respecto a la frecuencia y duración del tiempo que inviertes en los videojuegos?				
8. ¿Cuándo tienes problemas, usar los videojuegos te ayuda olvidarte de ellos?				
9. ¿Con qué frecuencia ignoras los pensamientos molestos sobre tu vida y los cambias por pensamientos agradables sobre los videojuegos?				
10. ¿Piensas que la vida sin videojuegos es aburrida, vacía y triste?				
11. ¿Te enfadas o te irritas, cuando alguien te molesta mientras juegas con algún videojuego?				
12. ¿Sufres alteraciones de sueño (te duermes tarde, se te quita el sueño) debido a aspectos relacionados con los videojuegos?				
13. ¿Cuándo no juegas con videojuegos te sientes agitado, preocupado o triste?				
14. ¿Sientes la necesidad de dedicarle cada vez más tiempo en los videojuegos para sentirte satisfecho?				
15. ¿Quitas importancia al tiempo que has estado jugando con videojuegos?				
16. ¿Dejas de salir con tus amigos para pasar más tiempo jugando con videojuegos?				
17. ¿Cuándo utilizas los videojuegos, se pasa el tiempo sin darte cuenta?				

Dependencia y Evasión (D y E): 1, 2, 3, 8, 10, 11, 15 y 16 = 32
Consecuencias Negativas (CN): 4, 5, 6, 7, 9, 12, 13, 14 y 17 = 36
Puntuación global = 68 puntos (a mayor puntuación mayor uso problemático con los videojuegos)
Nota. Ningún ítem se ha invertido
